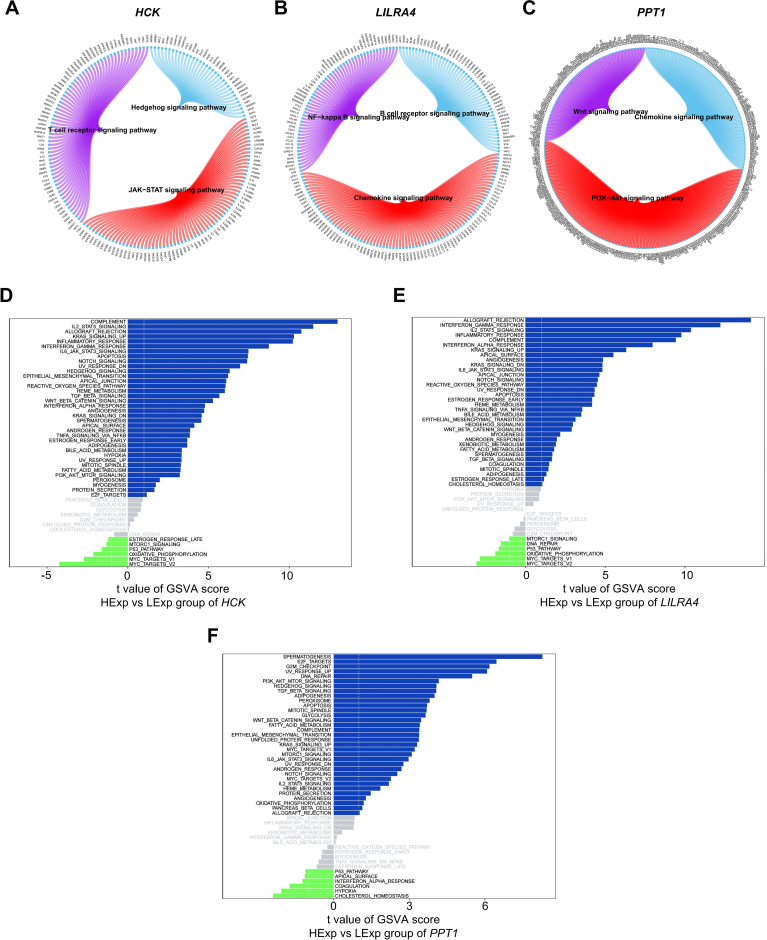# Deciphering the molecular landscape of oral squamous cell carcinoma: Novel biomarkers and therapeutic targets

**DOI:** 10.1016/j.gendis.2025.101552

**Published:** 2025-02-03

**Authors:** Qingyue Xiao, Kun Wang, Xiang Gao, Ping Ji, Jinlin Song

**Affiliations:** a College of Stomatology, Chongqing Medical University, Chongqing 401147, China; b Stomatological Hospital of Chongqing Medical University, Chongqing 401147, China; c Chongqing Key Laboratory of Oral Diseases, Chongqing 401147, China; d Chongqing Municipal Key Laboratory of Oral Biomedical Engineering of Higher Education, Chongqing 401147, China

Oral squamous cell carcinoma (OSCC) is one of the most common and aggressive types of oral cancer, accounting for over 90% of oral malignancies. Globally, the incidence of OSCC has been increasing, especially in regions with high rates of tobacco and alcohol consumption.^1^^,^^2^ In 2020, approximately 377,713 new cases of oral cancer were reported globally, with age-standardized incidence rates of 6.0 per 100,000 males and 2.3 per 100,000 females.^3^ Despite advances in treatment strategies, the prognosis for OSCC remains poor, as a significant proportion of cases are diagnosed at advanced stages, contributing to lower survival rates. Moreover, research on oral cancer lags behind that of other common cancer types globally.^4^ Consequently, the early detection and the identification of novel therapeutic targets are essential for improving clinical outcomes for OSCC patients.

This study employed multi-omics data analysis to systematically investigate the critical genes associated with OSCC and elucidate their potential molecular mechanisms. Gene expression data related to oral cancer were screened from the TCGA and GEO databases. Mendelian randomization and co-localization analyses were combined with expression quantitative trait loci (eQTL) and genome-wide association study (GWAS) databases for in-depth exploration. Single-cell and immune cell infiltration analyses were also performed to reveal the characteristics of the oral cancer microenvironment. This was followed by the construction of a nomogram model and drug sensitivity analysis to identify key biomarkers and potential therapeutic targets for personalized treatment strategies. The flow chart of the study design is presented in Figure S1.

Our analysis process began with Mendelian randomization on a training cohort to identify genes associated with OSCC risk. From a dataset of 315,025 oral cancer samples (314,193 controls and 832 cases), we identified 144 causal relationships between druggable genes and OSCC outcomes. Further filtering narrowed this to 73 gene pairs with positive eQTL outcomes, revealing 34 genes associated with a reduced OSCC risk and 39 genes linked to an increased risk (Fig. 1A and Table S1). Sensitivity analyses validated the robustness of these findings, as excluding any single SNP (single nucleotide polymorphism) did not significantly alter the overall results. We then analyzed oral cancer risk genes in the validation cohort, using summary statistics from 456,348 samples (456,192 controls and 156 cases) with outcome ID GCST90041793. Considering the module of genes corresponding to positive eQTL outcomes, Mendelian randomization identified four genes with significant associations (Fig. 1B; IVW *P*-value < 0.05). The genes *PPT1* (palmitoyl-protein thioesterase 1), *LILRA4* (leukocyte immunoglobulin-like receptor A4), and *HCK* (hemopoietic cell kinase) were correlated with a lower risk of oral cancer, while *TNFRSF13C* (tumor necrosis factor receptor superfamily member 13c) was linked to a higher risk. A sensitivity analysis of these four genes further validated the causal relationships, demonstrating that excluding any single SNP did not significantly alter the confidence intervals, confirming the robustness of the results (Fig. 1C). Co-localization analysis at the eQTL-GWAS level revealed that *HCK*, *LILRA4*, and *PPT1* had a co-localization probability (SNP.PP.H4) greater than 0.7, indicating strong associations between these genes and OSCC (Fig. 1D and Table S2). Therefore, the three genes *HCK*, *LILRA4*, and *PPT1*, were prioritized for further investigation.

**Figure 1:**
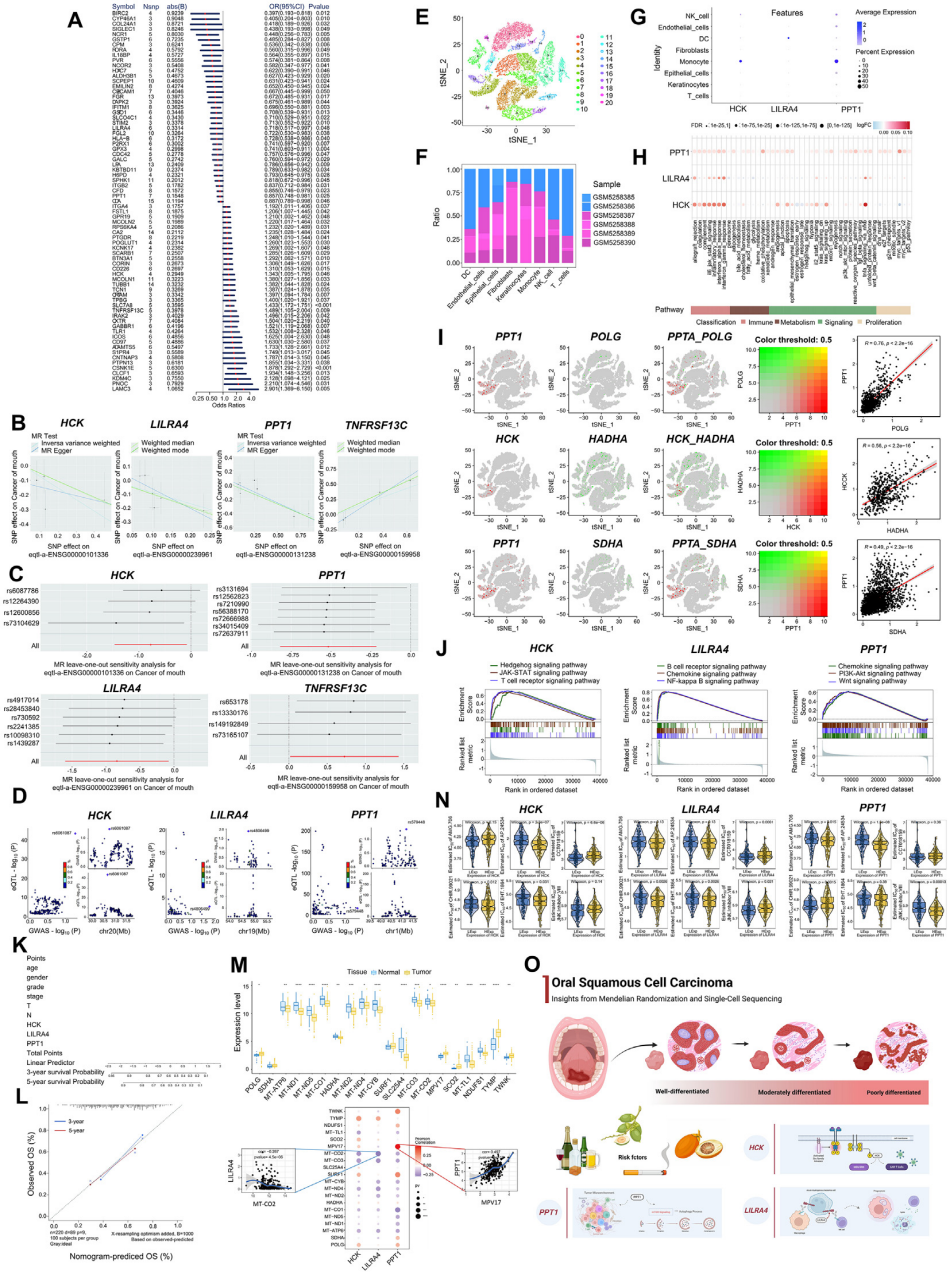
Molecular landscape of oral squamous cell carcinoma: novel biomarkers and therapeutic targets. **(A)** Mendelian randomization analysis identified 73 pairs of causal risk. **(B)** Scatter plots of Mendelian randomization (MR) analysis of important genes in the validation set. **(C)** Forest plot of the leave-one-out test for single nucleotide polymorphisms (SNPs) associated with important genes in the validation set. **(D)** Associations between SNPs in key genes and diseases. **(E)** Cell annotation of 21 clusters. **(F)** Differences in the proportion of 8 cell types between the two sample groups. **(G)** Expression of key genes in single cells. **(H)** Correlation of key genes with immunometabolism. **(I)** Gene co-expression of mitochondria-related genes with key genes in single-cell data and the co-expressed gene correlations. **(J)** Gene set enrichment analysis (GSEA) of critical genes. **(K**–**M)** Correlation between nomogram model construction and tumor progression genes. **(N)** Drug sensitivity correlation of key genes. **(O)** Graphical abstract.

To investigate the expression profiles and mechanistic pathways of these key genes in OSCC, we analyzed single-cell sequencing data from GSE172577, comprising a total of six samples. Following stringent data filtering (nFeature_RNA > 500 and percent.mt < 5), the top 10 genes with the highest variability were identified (Fig. S2A–C). The subsequent data processing included standardization, normalization, and principal component analysis (Fig. S2D). T-distributed stochastic neighbor embedding analysis delineated 21 distinct cell subpopulations, categorized into 8 major cell types (Fig. 1E; Fig. S2E). The cell proportion distribution was presented in Figure 1F, and gene expression patterns across these cell types revealed significant differences (Fig. 1G; Fig. S2F). AUCell analysis provided insights into the immune and metabolic pathway interactions of these key genes (Fig. 1H). Mitochondrial function-related genes were further investigated using the GeneCards database. The top three genes *POLG* (DNA polymerase gamma), *SDHA* (succinate dehydrogenase complex flavoprotein subunit A), and *HADHA* (hydroxyacyl-CoA dehydrogenase trifunctional multienzyme complex subunit alpha) were co-expressed with the three key genes identified in our study, visualizing their interactions within the eight-cell categories (Fig. 1I; Fig. S3). Next, we analyzed the immune microenvironment in OSCC, using a dataset from the TCGA database with 286 samples (19 normal samples and 267 disease samples). Immune cell percentages were quantified, revealing significant differences in immune functions such as antigen-presenting cell co-inhibition and parainflammation between normal and disease groups (Fig. S4A–C). Notably, *HCK* was strongly associated with T helper cells, *LILRA4* with plasmacytoid dendritic cells, and *PPT1* with macrophages, indicating their roles in immune cell infiltration (Fig. S5A). An analysis using the TISIDB database confirmed the involvement of these key genes in immune regulation, interactions with immune regulatory factors, and chemokines (Fig. S5B). To further explore the contribution of the key genes to OSCC progression, we conducted gene set enrichment analysis (GSEA) and gene set variation analysis (GSVA). GSEA revealed that *HCK* participated in the Hedgehog and JAK-STAT signaling pathways, *LILRA4* in the B cell receptor and chemokine signaling pathways, and *PPT1* in the chemokine and PI3K (phosphoinositide 3-kinase)-AKT (protein kinase B) pathways (Fig. 1J; Fig. S6A–C). GSVA showed that high *HCK* expression was linked to TGF-β (transforming growth factor-beta) and Wnt-β-catenin signaling (Fig. S6D), *LILRA4* was associated with the Notch and reactive oxygen species pathways (Fig. S6E), and *PPT1* was involved in the PI3K-AKT-mTOR (mammalian target of rapamycin) and Hedgehog pathways (Fig. S6F).

To predict OSCC prognosis, we constructed a nomogram model integrating key genes and clinical indicators (Fig. 1K). The model accurately predicted three- and five-year overall survival, as confirmed by close agreement with observed outcomes (Fig. 1L). Additionally, we identified 20 disease-related genes, with *POLG*, *MT-ATP6* (mitochondrially encoded ATP synthase membrane subunit 6), and *MT–ND1* (mitochondrially encoded NADH:ubiquinone oxidoreductase core subunit 1) showing differential expression between patient cohorts. Correlations were found between key genes and disease-regulated genes, such as *PPT1* and *MPV17* (*r* = 0.497), and *LILRA4* and *MT–CO2* (mitochondrially encoded cytochrome C oxidase II) (*r* = −0.267; Fig. 1M). Finally, to assess the relationship between key genes and chemotherapy responsiveness, we conducted drug sensitivity analyses using the GDSC database and the “pRRophetic” R package. Our results showed that *HCK* was associated with sensitivity to drugs such as AP24534, CCT018159, CHIR99021, and EHT1864. *LILRA4* was linked to AMG706, CCT018159, CHIR99021, EHT1864, and JNK inhibitor VIII, while *PPT1* was associated with AMG706, AP24534, CHIR99021, and JNK inhibitor VIII (Fig. 1N). These findings underscore the potential of these key genes to influence chemotherapy effectiveness, laying the foundation for personalized treatment in early-stage OSCC.

In conclusion, oral cancer remains a significant global health concern due to its high prevalence and poor prognosis. A comprehensive understanding of its epidemiology, genetic markers, and immune dynamics is essential for improving patient outcomes. Key genes such as *HCK*, *LILRA4*, and *PPT1* play critical roles in the disease’s pathogenesis, offering promising therapeutic targets (Fig. 1O). Future research is expected to focus on validating early diagnostic biomarkers and corresponding therapeutic targets. Combining traditional therapies with targeted treatments and immunotherapies holds significant promise for improving the prognosis and quality of life for oral cancer patients.^5^ Advances in bioinformatics will continue to unravel deeper insights into the molecular mechanisms driving oral cancer, thus leading to more precise and personalized treatment approaches.

## Funding

This study was supported by grants from the Chongqing Municipal Postdoctoral Science Foundation (China) (No. CSTB2023NSCQ-BHX0033) and the Program for Youth Innovation in Future Medicine of Chongqing Medical University (Chongqing, China) (No. W0055).

## CRediT authorship contribution statement

**Qingyue Xiao:** Writing – original draft, Investigation, Funding acquisition, Formal analysis, Conceptualization. **Kun Wang:** Methodology, Investigation. **Xiang Gao:** Writing – review & editing, Project administration, Funding acquisition, Data curation. **Ping Ji:** Visualization, Supervision. **Jinlin Song:** Supervision, Conceptualization.

## Conflict of interests

The authors of this manuscript affirm that they have no known competing financial interests or personal relationships that could have influenced the work reported in this paper. A thorough review of all affiliations has been conducted, and no potential conflict of interests has been identified that could be perceived as affecting the integrity of the research.

## Figures and Tables

**figs1:**
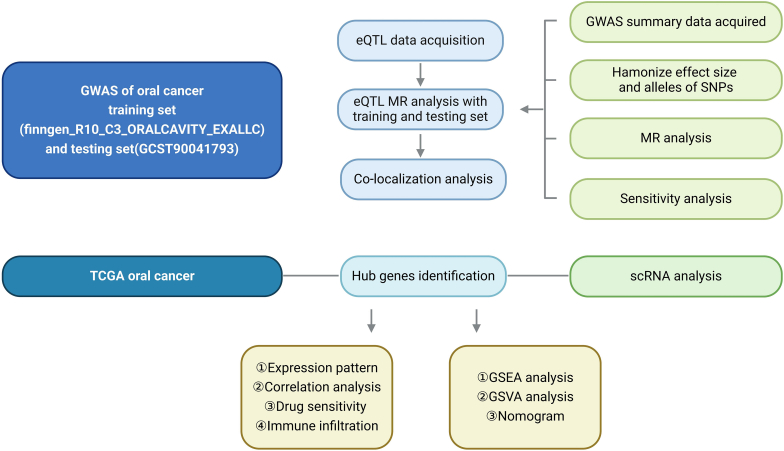


**figs2:**
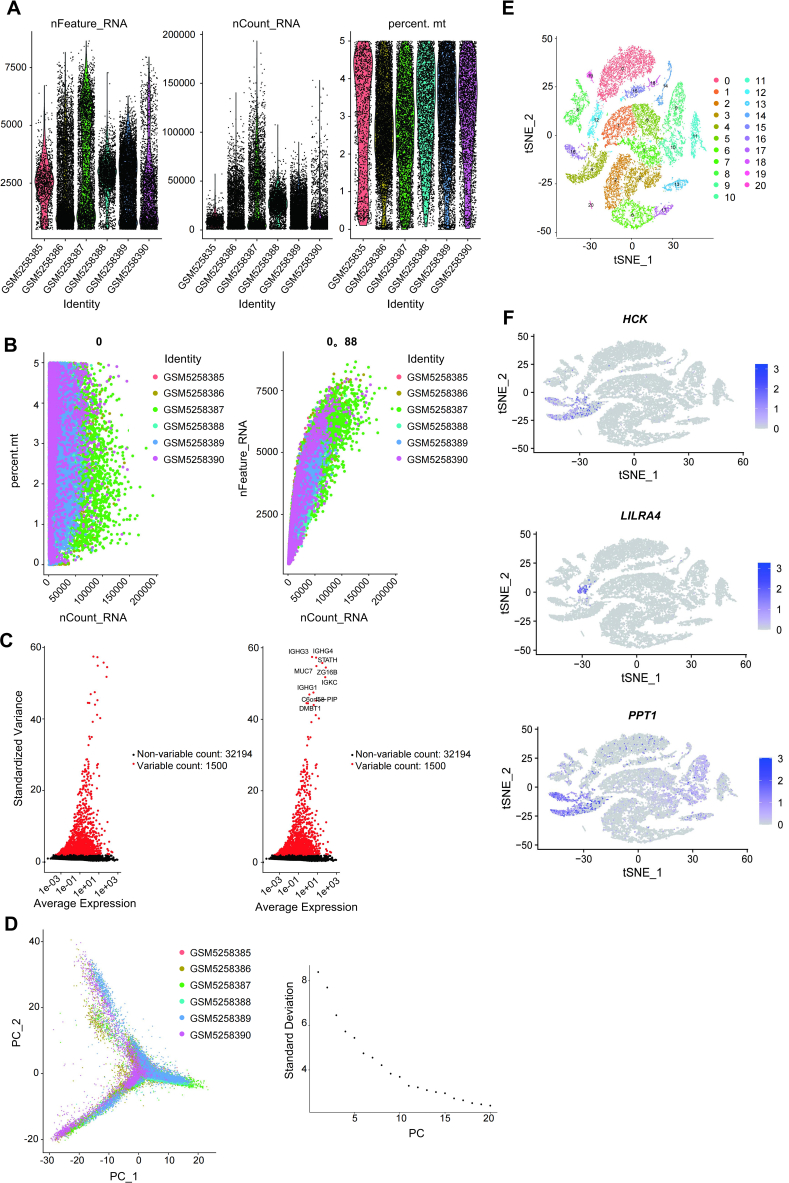


**figs3:**
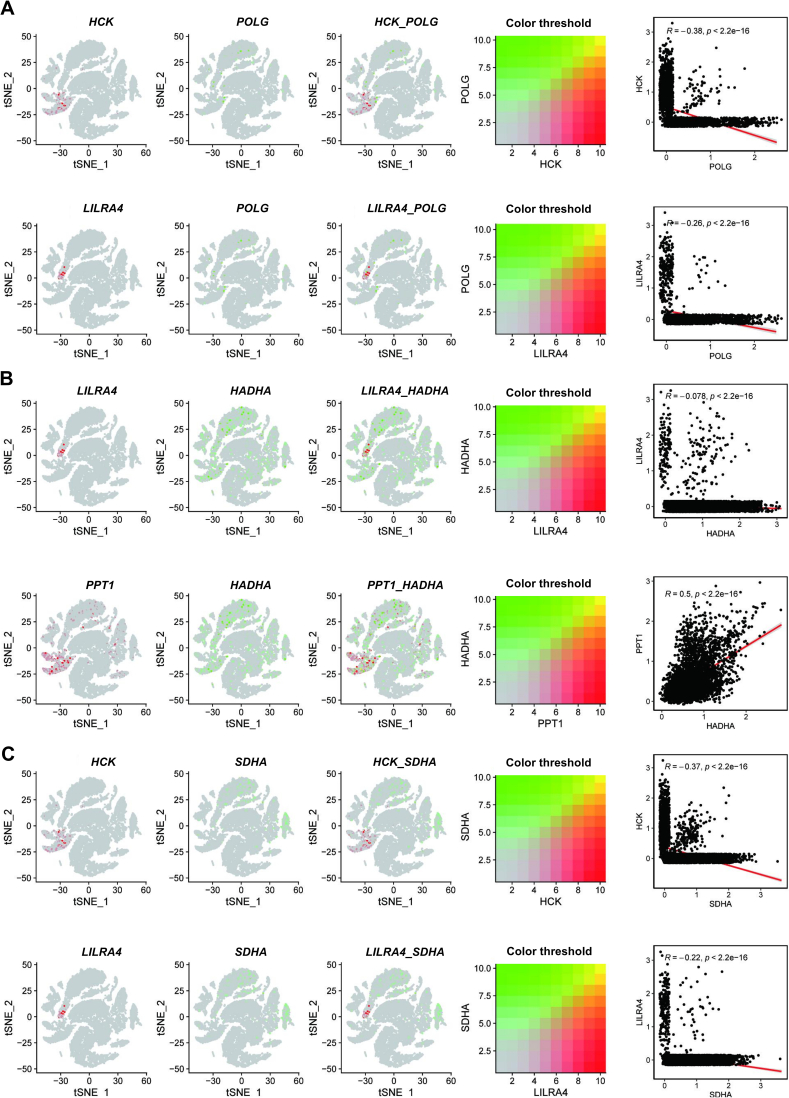


**figs4:**
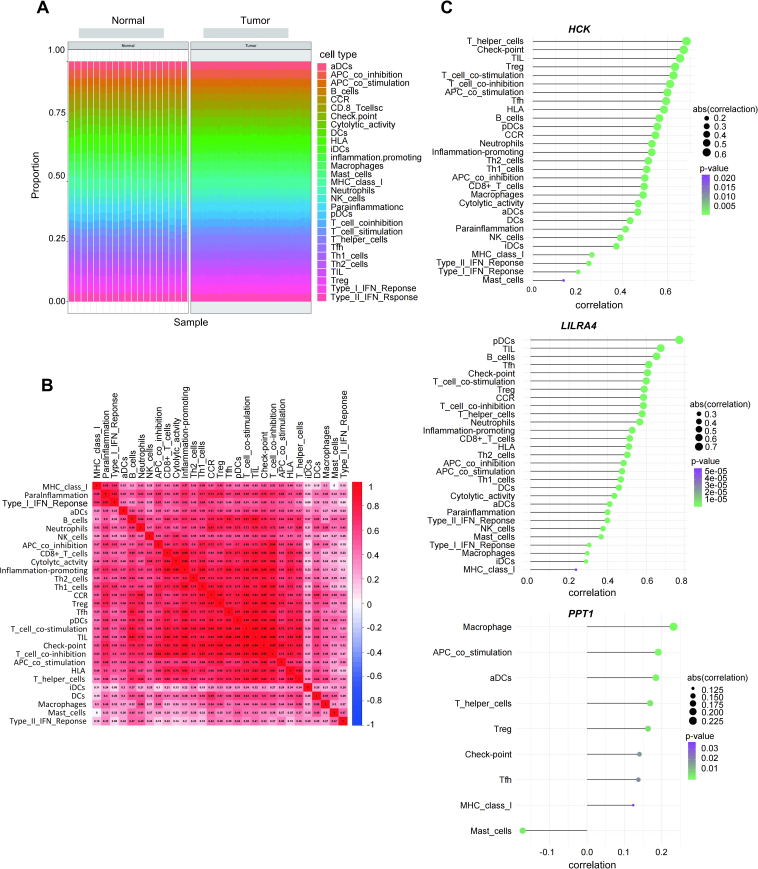


**figs5:**
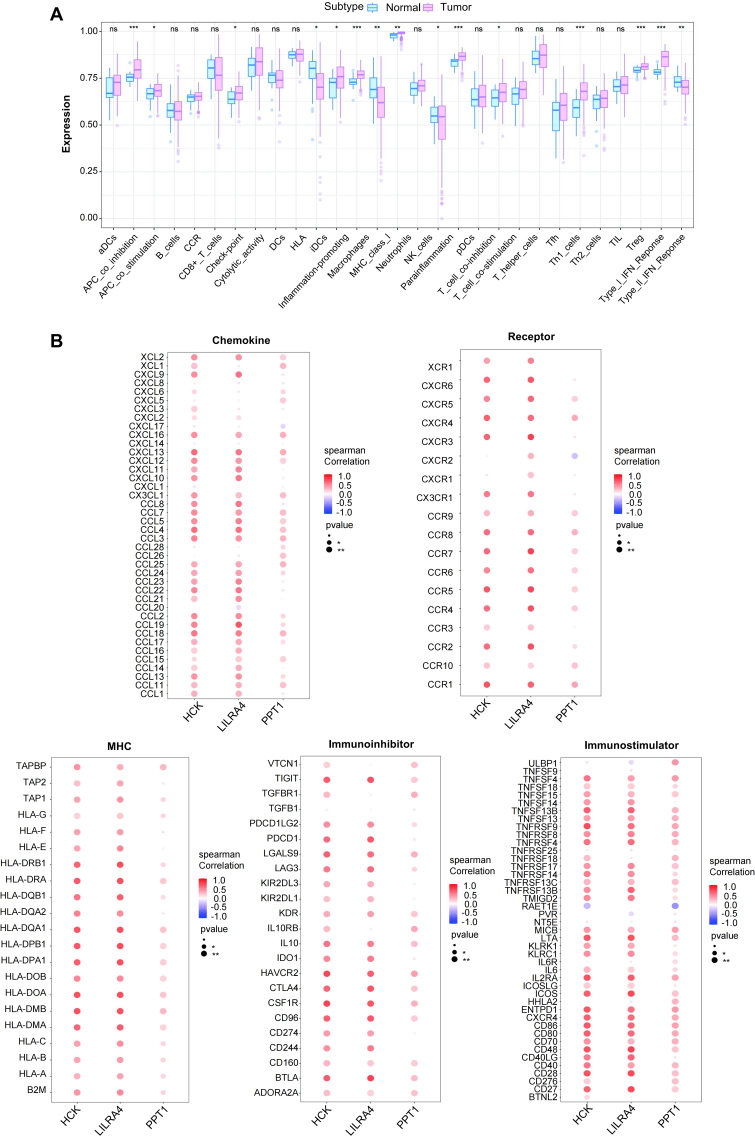


**figs6:**