# Supplementary material for: Deciphering the molecular landscape of oral squamous cell carcinoma: Novel biomarkers and therapeutic targets
Source: Genes Dis. 2025 Feb 3;12(5):101552. doi: 10.1016/j.gendis.2025.101552 (PMC12166728; doi:10.1016/j.gendis.2025.101552)
Supplement: Multimedia component 1 [file mmc1.docx]

**Supplementary Information**

**Deciphering the Molecular Landscape of Oral Squamous Cell Carcinoma: Novel Biomarkers and Therapeutic Targets**

**1. Important Materials and MethodsAbstract**

1.1 Data acquisition

The TCGA database (https://portal.gdc.cancer.gov/) was one of the most comprehensive cancer gene information databases, containing gene expression data, miRNA expression data, lncRNA expression data, copy number variations, DNA methylation, SNPs, and more. The expression data for OSCC included 19 samples from the normal group and 267 samples from the tumor group.

The GEO database (Gene Expression Omnibus) was a resource managed by the National Center for Biotechnology Information (NCBI) for storing gene expression data, with address at https://www.ncbi.nlm.nih.gov/geo/info/datasets.html. We downloaded the single-cell data file GSE172577 from this public database, which included comprehensive expression profile data from 6 patients.

The eQTL data were sourced from the eQTLGen consortium database (https://www.eqtlgen.org). The eQTLGen consortium aimed to elucidate the genetic structure of blood gene expression and comprehend the complex traits. Currently, in its second phase, the large-scale eQTLGen project was dedicated to performing extensive genome-wide meta-analyses in blood samples.

The outcome data connected to GWAS studies primarily involved participants of European ancestry. The aggregated data for the training set outcomes were obtained from the FinnGen database. FinnGene database was a genetic research database focusing on European populations, aiming to study unique genetic diseases and mutations in European populations. It contained data from many samples from different regions, highlighting the links between genes and diseases. This database was particularly important for understanding the use of genetics in public health, particularly in disease prevention and personalized medicine. Among them, oral cancer (finngen_R10_C3_ORALCAVITY_EXALLC) had 832 cases and 314,193 controls. The validation set outcome summary data came from the GWAS database (GCST90041793). The GWAS was aligned with Genome Assembly and dbSNP Build. For oral cancer, there were 156 cases and 456,192 controls included in the analysis.

1.2 Mendelian randomization analysis

Outcome IDs were selected from the FinnGen, and GWAS databases were obtained through GWAS summary data (https://gwas.mrcieu.ac.uk/). In the eQTL, the relevant causal relationships were subsequently identified. At a significance threshold of P < 1e-6, the SNPs associated with each gene were used as possible instrumental variables (IVs). Then, linkage disequilibrium (LD) was detected, and only R2 < 0.001 (clumping window size) was retained = 10,000kb snp, while weak instrumental variables were filtered out using an F value > 10. The analysis sequentially used several methods: Inverse Variance Weighted (IVW), which integrated the Wald estimates with meta-analysis; MR Egger, which operated under the premise that the instrument's strength was not influenced by its direct impacts; Weighted Median, which could accurately determine the causality of cases with invalid IVs in up to 50%; Weighted Mode, which demonstrated a stronger ability to estimate causal effects compared to MR-Egger regression, with reduced bias and Type I error rates. The causal relationship was assessed via two statistical approaches (if only one method was available for the SNP in question, the Wald ratio was adopted) to derive an awareness of the effect of cis- and certain cross-regional gene expression with oral cancer in whole-blood.

1.3 Sensitivity analysis

The leave-one-out sensitivity analysis of Mendelian Randomization was performed to assess the effect of individual genetic variants on the risk of developing oral cancer. This approach systematically excluded each SNP at a time and recalculated the pooled effect size for the remaining SNPs. Excluding each SNP generated a new point estimate, and its 95% confidence interval enabled the assessment of each SNP's distinct contribution and the robustness of the overall findings. Estimates after the removal of individual SNPs were summarized, along with the overall estimates when all SNPs were calculated. By comparing these values, the impact of excluding any single SNP on the overall results could be observed, thus evaluating the robustness of our analysis.

1.4 Colocalization analysis

We applied the coloc method to perform the colocalization analysis, along with eQTL summary data and GWAS for oral cancer. Moreover, we estimated the posterior probability with a 100-kilobase region surrounding the index SNP. In the colocalization analysis, H3 indicated the posterior probability that gene expression and oral cancer were associated but had different causal variants, while H4 indicated the posterior probability of the two traits associated with the single causal variant shared. A threshold of SNP.PP.H4 > 0.7 was set for colocalization.

1.5 Single-cell analysis

The expression profile was first screened by employing the Seurat package. Based on criteria (nFeature_RNA > 500 & percent.mt < 5), the genes with low expression were eliminated. Next, we sequentially applied standardization, normalization, PCA, as well as subsequent other analyses with the data. This was followed by the observation of the optimal pc number through ElbowPlot. The positional relationship was determined via TSNE analysis in each cluster. In addition, the cell dex package was used for annotation, identifying cells that played significant roles in tumor development.

1.6 Immune cell infiltration analysis

The ssGSEA method was commonly utilized to assess the types of immune cells involved in the tumor microenvironment. In this research, we employed the ssGSEA algorithm to illustrate the expression profile of immune cells and estimated the relative percentage of immune infiltrating cells with different types.

1.7 GSEA analysis

Based on gene expression levels, patients were categorized into two groups. GSEA was then employed to analyze variations in signaling pathways with different groups. The background gene set, used for annotating subtype pathways, was sourced in the Molecular Signatures Database (MsigDB) database, and the detection of differential pathway expression between subtypes was conducted. Gene sets with significant enrichment were identified with a consistency score, where an adjusted p-value below 0.05 was regarded as significant. In short, GSEA analysis was frequently utilized in research to integrate disease classification with biological significance.

1.8 GSVA analysis

For assessing the enrichment of gene sets in transcriptomic data, gene set variation analysis (GSVA) was an unsupervised, non-parametric approach. GSVA transformed gene-level variations into pathway-level alterations by evaluating the gene sets of interest, hence elucidating the biological functions. In the research, gene sets were downloaded from version 7.0 of MsigDB, and the GSVA algorithm was employed to thoroughly assess each gene set and examine potential changes across samples in biological functions.

1.9 Nomogram model construction

The nomogram was developed using regression analysis, integrating gene expression and clinical symptoms. Scaled line segments for each variable were plotted on the same plane, illustrating their combined effects and interactions within the prediction model. The approach of multifactor regression analysis assigned scores to each value level with influencing factors based on their contributions to the outcome variable. These individual scores were summed to produce a total score, which was then used to calculate the predicted value.

1.10 Drug sensitivity analysis

Using data from the GDSC Cancer Drug Sensitivity Genomics Database, which was known as the comprehensive pharmacogenomics database at https://www.cancerrxgene.org/, we employed the R package “pRRophetic” with each tumor sample to forecast the chemotherapy sensitivity. Regression methods were utilized to estimate the IC50 for specific chemotherapy treatments. With the GDSC training set, a 10-fold cross-validation was conducted to confirm the accuracy of the regression and prediction results. Default parameters were used for whole settings, applied "combat" to correct for batch effects, as well as equalization for duplicate gene expressions.

1.11 Statistical analysis

Reliable MR analysis relied on three foundational assumptions: (1) Correlation assumption (instrumental variables were closely linked to the exposure, without direct influence towards the outcome); (2) Independence assumption (instrumental variables were unrelated to confounding factors); (3) Exclusion assumption (instrumental variables should influence the outcome solely through the exposure. Once the outcome was affected by an instrumental variable via other pathways, it indicated the presence of gene pleiotropy). For this analysis, R language (version 3.0) was utilized. All statistical tests were performed as two-sided, with a p-value of below 0.05 regarded as statistically significant.

**2. Additional Results**

The flow chart outlining the design of this study was demonstrated in **Fig. S1A**.

2.1 Mendelian randomization analysis of training cohort to obtain oral cancer risk genes

We obtained the druggable genes from the literature,**^1^** and acquired the outcome ID via the summary statistics of 315,025 oral cancer-related samples (Controls: 314,193; Cases: 832): finngen_R10_C3_ORALCAVITY_EXALLC. Using extract_instruments and extract_outcome_data successively, 144 pairs of causal relationships were extracted considering the genes and outcomes. The causal relationships of the 73 pairs of module genes associated with eQTL positive outcomes were further screened through Mendelian randomization analysis (IVW pval < 0.05). Genes *BIRC2*, *CYP46A1*, *COL24A1*, *SIGLEC1*, *NCR1*, *GSTP1*, *CPM*, *RORA*, *IL18BP*, *PVR*, *NCOR2*, *HDAC7*, *ALDH3B1*, *SCPEP1*, *EMILIN2*, *CEACAM1*, *FGR*, *DAPK2*, *IFITM1*, *GSTO1*, *SLCO4C1*, *STIM2*, *LILRA4*, *FGL2*, *HLA−B*, *P2RX1*, *GPX3*, *CDC42*, *GALC*, *LIPA*, *KBTBD11*, *H6PD*, *SPHK1*, *ITGB2*, *CFD*, *PPT1*, *CDA* were associated with low risk of oral cancer; genes *ITGA4*, *FSTL1*, *GPR19*, *MCOLN2*, *RPS6KA4*, *CA2*, *PTGDR*, *POGLUT1*, *KCNK17*, *SCARB1*, *BTN3A1*, *CORIN*, *CD226*, *HCK*, *MCOLN1*, *TUBB1*, *TCN1*, *CRTAM*, *TPBG*, *SLC7A8*, *TNFRSF13C*, *IRAK2*, *OXTR*, *GABBR1*, *TLR1*, *ICOS*, *CD97*, *ADAMTS5*, *S1PR4*, *CNTNAP3*, *PTPN13*, *CSNK1E*, *CLCF1*, *KDM4C*, *PNOC*, and *LAMC3* were associated with a higher risk of oral cancer (**Table S1**). To further ensure the reliability of the causal relationships identified with the 73 genes, additional sensitivity analyses were performed. The outcome demonstrated that excluding any single SNP had no major influence on the overall error bars, indicating the robustness of the causal relationships within 73 pairs we selected.

2.2 Analysis of oral cancer risk genes in the validation cohort

Then, through the summary statistics of 456,348 oral cancer-related samples in the validation set (Controls: 456,192; Cases: 156), the outcome ID was obtained: GCST90041793. According to extract_instruments and extract_outcome_data in sequence, 73 pairs of causal relationships between module genes and outcomes were extracted. Considering module genes corresponding to the positive outcome of eQTL, the causal relationship of 4 pairs was screened out with Mendelian randomization analysis (IVW pval < 0.05). The relevant genes were *HCK* (hemopoietic cell kinase), *LILRA4* (leukocyte immunoglobulin-like receptor A4), *PPT1* (palmitoyl-protein thioesterase 1), and *TNFRSF13C*. Genes *PPT1* (0.596; 0.441−0.807; P = 0.001), *LILRA4* (0.432; 0.202−0.925; P = 0.031), *HCK* (0.462; 0.231−0.923; P = 0.059) were correlated with a low risk of oral cancer; gene *TNFRSF13C* (2.066; 1.017–4.193; P = 0.045) was linked to a higher risk of oral cancer.

2.3 Co-localization analysis to identify the critical genes as *HCK*, *LILRA4*, and *PPT1*

We conducted an in-depth sensitivity analysis on the causal relationship to confirm the reliability of the 4 genes. The findings illustrated that excluding any one SNP did not significantly affect the overall error bar, suggesting the robustness of the selected 4 pairs of causal relationships. Meanwhile, co-localization analysis was performed on 4 genes at the eQTL-GWAS level, among which the co-localization SNP.PP.H4 of genes *HCK*, *LILRA4*, and *PPT1* was higher than 0.7. The key genes were identified as *HCK*, *LILRA4*, and *PPT1*, which were the primary focus of further investigation and analysis (**Table S2**).

2.4 Single-cell sequencing revealed the key gene expression profiles in oral cancer

To investigate the high-dimension expression profile as well as mechanistic pathways of the key genes, the single-cell data from GSE172577 were downloaded, comprising a total of 6 samples. Based on nFeature_RNA and nCount_RNA criteria (nFeature_RNA > 500 & percent.mt < 5), the data samples were initially filtered (**Fig. S2A, B**). Following this initial process, the 10 genes with the highest level of deviation were highlighted (**Fig. S2C**). The data were processed in sequence through standardization, homogenization, and PCA analysis (**Fig. S2D**). Then, the TSNE analysis was performed, and 21 distinct subgroups were identified (**Fig. S2E**). Our study proceeded to classify each identified subtype and grouping them into 8 cell categories: T cells, Keratinocytes, Epithelial cells, Monocytes, Fibroblasts, Dendritic cells (DC), Endothelial cells, and Natural Killer (NK) cells showing the cell proportion histogram of these 8 cells. We also examined the expression levels of key genes across the 8 cell categories at the single-cell level (**Fig. S2F**). Additionally, AUCell was employed to determine immune and metabolic pathway activities, enabling a comprehensive awareness of the interaction of the key genes with these aspects. Using the GeneCards database (https://www.genecards.org/), we retrieved the index term "mitochondrial function" to acquire mitochondrial function-related genes and selected the top three genes according to the relevance scores. The mitochondria-related regulatory genes (*POLG*, *SDHA*, *HADHA*) and the three key genes were co-expressed in 8 types of cells to visualize the gene co-expression (**Fig. S3**).

2.5 OSCC data analysis suggested the impacts of key genes on immune microenvironment and cell infiltration

We downloaded the OSCC data set from the TCGA public database for function analysis, which included a total of 286 sample data, comprising 19 cases from the normal cohort and 267 cases from the disease cohort. The immune microenvironment consisted of a complex network of immune cells, cytokines, and chemokines.**^2^** These components played a crucial role in both the pathology and therapeutic strategies for oral cancer. Our study further aimed to elucidate how key genes affected the development of oral cancer at the molecular level, from the association between these genes and immune cell infiltration within the oral cancer dataset. We quantified the percentage of immune cells in samples and exhibited the relationship among different immune cell types (**Fig. S4A, B**). In addition, the research demonstrated that immune cells, such as APC_co_inhibition and parainflammation, were important between the two groups (**Fig. S4C**). This study then uncovered the interplay between the key genetic factors and immune cells (**Fig. S5A**). Among them, *HCK* showed a strong positive correlation with T helper cells, etc. *LILRA4* was positively correlated with plasmacytoid dendritic cells (pDC), etc., while *PPT1* exhibited a significant positive association with Macrophages, etc. Moreover, the associations between the focused genes and various immune factors were analyzed using data from the TISIDB database, considering immune regulatory factors, cell receptors, and chemokines (**Fig. S5B**). The results indicated that the 3 genes we focused on were associated with immune cell infiltration, taking an active part in immune response dynamics.

2.6 Molecular pathways associated with the critical genes in disease progression

To uncover the molecular processes through which the three primary genes affect disease progression, we further performed the GSEA analysis. The results revealed that *HCK* was involved in several enriched pathways, including the Hedgehog signaling pathway and the JAK-STAT signaling pathway. (**Fig. S6A**). *LILRA4* actively participated in the pathways, such as the B cell receptor and chemokine signaling pathway (**Fig. S6B**). Additionally, *PPT1* was closely linked to the chemokine signaling pathway and the PI3K-AKT signaling pathway. (**Fig. S6C**). GSVA analysis showed signaling pathways activated by high gene expression levels. For *HCK*, such as TGF-beta signaling and Wnt-beta-catenin signaling were activated (**Fig. S6D**); *LILRA4*, like Notch signaling and the Reactive oxygen species pathway, were enriched (**Fig. S6E**); *PPT1*, such as PI3K-AKT-mTOR signaling and Hedgehog signaling pathways were actively participated (**Fig. S6F**).

2.7 Nomogram-based regression analysis and prognostic evaluation of key genes in OSCC

We illustrated the regression analysis results using a nomogram with the expression levels of the critical genes. The results demonstrated across all samples that clinical indicators of oral cancer and the expression distribution of critical genes contributed at varying degrees in the overall scoring process. Besides, we performed prognostic analysis for overall survival (OS) at three-year and five-year intervals. The outcome indicated that the predicted OS was closely aligned with the observed OS, suggesting the predictive accuracy of the nomogram model. Moreover, we distinguished the disease-related regulatory genes using the GeneCards database (https://www.genecards.org/). We examined the 20 target genes with the top relevance scores and found genes such as *POLG*, *MT-ATP6*, and *MT-ND1* exhibited differential expressions in the two patient cohorts. We also performed an association analysis between essential genes and disease-regulated genes. Our results showed significant correlations in their expression levels. Notably, *PPT1* exhibited a positive association with *MPV17* (r=0.497), while *LILRA4* illustrated the inverse correlation with *MT-CO2* (r=−0.267).

2.8 The modulate chemotherapy sensitivity of key genes in early-stage with OSCC

The combination of surgery and chemotherapy has proven effective for treating early-stage oral cancer. We further searched in GDSC database for the drug sensitivity data and employed the “pRRophetic” R package to estimate the chemotherapy sensitivity. We investigated the association between key genes and their responsiveness to commonly used chemotherapeutic drugs. The research findings revealed that *HCK* was associated with sensitivity to drugs such as AP.24534, CCT018159, CHIR.99021, and EHT.1864. *LILRA4* showed a close correlation with the sensitivity to AMG.706, CCT018159, CHIR.99021, EHT.1864, and JNK Inhibitor VIII. *PPT1* was linked to the sensitivity to AMG.706, AP.24534, CHIR.99021, and JNK Inhibitor VIII. These results highlighted the regulation of these genes in influencing the effectiveness of specific chemotherapeutic agents, paving the way for personalized treatment strategies.

**3. Discussion on additional results**

Oral cancer is globally recognized as a kind of malignant tumor with high morbidity and mortality. It is notably prevalent in regions such as Southeast Asia, South Asia, and Eastern Europe.**^3^** The timely identification and intervention for oral cancer are essential for improving patient survival outcomes. The biomarkers have the potential to forecast disease progression, inform personalized therapeutic strategies, and evaluate the prognostic landscape of patients with this condition. Our study on the biomarkers of OSCC is expected to contribute to the construction of the risk score system, focusing on the identification of pivotal genes that play a crucial role in the clinical management of oral cancer.**^4,5^**

Empirical evidence has underscored the pivotal influence that immune cell infiltration exerts in the progression of oral cancer. This process involves the migration and accumulation of various immune cell types, which are essential in modulating the tumor behavior and the response of the host to the malignancy.**^6,7^** The degree of immune infiltration is recognized as a significant factor that influences both disease progression and patient prognosis. During the early phases of oral cancer, the immune system is capable of discerning and eliminating tumor cells.**^8,9^** However, as the malignancy progresses and the tumor microenvironment undergoes transformation, the function of immune cells can be compromised, and tumor cells may evade immune surveillance through a spectrum of adaptive mechanisms.**^10^** The modulation of immune checkpoint molecules, such as the PD-1/PD-L1 axis and CTLA-4, is crucial in the phenomenon of immune escape.**^11,12^** Furthermore, the enrichment of regulatory T cells (Tregs) and myeloid-derived suppressor cells (MDSCs) within the tumor microenvironment correlates with an enhanced state of immunosuppression and diminished responsiveness to therapeutic interventions.**^13^** Immunotherapy as a promising therapeutic approach, aims to enhance the intrinsic capabilities of the patient's immune system to identify and attack cancer cells.**^14^** The advances in single-cell sequencing and sophisticated bioinformatics techniques have enabled a deeper exploration of the cellular dynamics within the tumor microenvironment during the process of oral carcinogenesis. Collectively, these studies suggest the potential of immunotherapy to improve the survival and quality of life of oral cancer patients.**^15,16^**

Among the three pivotal genes we have identified, *HCK* is a non-receptor tyrosine kinase belonging to the Src family, which could facilitate the recruitment of inflammatory phagocytic cells, thereby enhancing phagocytosis and the formation of podosome rosettes.**^17,18,19^** In the tumor microenvironment, activation of *HCK* expression promotes the adhesion of tumor cells to the extracellular matrix and enhances tumor cell invasiveness, which in turn promotes tumor spread and metastasis.**^20^** In addition, *HCK* is associated with tumor angiogenesis, which increases tumor blood supply by promoting endothelial cell proliferation and migration, providing nutrients and oxygen for tumor growth.**^21,22,23^** Our study provided data to support the potential of *HCK* as a biomarker of tumor progression and as a therapeutic target. *LILRA4* is a member of the immune globulin superfamily expressed predominantly on myeloid cells, particularly in monocytes and dendritic cells.**^24,25^** *LILRA4* is known  for encoding the immunoglobulin-like cell surface protein predominantly on pDCs as the surface receptor.**^26^** It typically serves as a negative regulatory receptor on pDC in a resting state. At the same time, in the condition of bacterial or viral infection, its expression is significantly reduced, thus modulating the function of cells in the immune response. Additionally, *LILRA4* has been identified as a potential biomarker associated with non-small cell lung cancer (NSCLC) metastasis.**^27,28,29^** *PPT1* plays a significant role in regulating protein stability and cellular signaling through the catalytic removal of palmitic acid from proteins.**^30,31^** It is revealed that in hepatocellular carcinoma, *PPT1*-positive macrophages are closely associated with immunosuppressive contexture as well as immunotherapy response.**^32^** *PPT1* could regulate cellular iron death by affecting the palmitoylation of GPX4, thus stimulating the growth and restraining the ferroptosis within OSCC cells.**^30^** In addition, a first-in-human trial of *PPT1* inhibition in patients with primary and secondary hepatocellular carcinoma using oral treatment with GNS561/Ezurpimtrostat has been initiated, and outcomes from this project are awaited.**^33^**

Research on antitumor agents in the field of oral cancer treatment continues to make progress, with the goal of improving therapeutic efficacy and patient survival.**^34^** By analysing the genomic features of tumors, the development of targeted therapeutic agents against specific gene targets provides more precise therapeutic options, which are expected to improve the quality of life for patients.**^35^** Therefore, an in-depth study of the molecular mechanisms and key genes of oral cancer is of great value for disease prevention, diagnosis, treatment, and prognosis assessment.

**Data Availability Statement:** Transcriptome data of TCGA-OSCC were from the TCGA database (https://portal.gdc.cancer.gov/). The single-cell transcriptome was accessible with the GEO database (https://www.ncbi.nlm.nih.gov/geo/query/acc.cgi?acc=GSE172577). The eQTL data were available in the eQTLGen Consortium (https://www.eqtlgen.org). The GWAS data could be found in the IEU Open GWAS Project (https://gwas.mrcieu.ac.uk/).

**Acknowledgments:** We express our gratitude to the participants and researchers for their contribution to the FinnGen and GWAS Catalog.

**References**

1. Finan C, Gaulton A, Kruger FA, et al. The druggable genome and support for target identification and validation in drug development. Sci Transl Med. 2017;9(383).
2. Yuan Y, Jiang YC, Sun CK, Chen QM. Role of the tumor microenvironment in tumor progression and the clinical applications (Review). Oncol Rep. 2016;35(5):2499-2515.
3. Goud E, Malleedi S, Ramanathan A, et al. Association of Interleukin-10 Genotypes and Oral Cancer Susceptibility in Selected Malaysian Population: A Case- Control Study. Asian Pac J Cancer Prev. 2019;20(3):935-941.
4. Viet CT, Yu G, Asam K, et al. The REASON score: an epigenetic and clinicopathologic score to predict risk of poor survival in patients with early stage oral squamous cell carcinoma. Biomark Res. 2021;9(1):42.
5. Kinane DF, Gabert J, Xynopoulos G, Guzeldemir-Akcakanat E. Strategic approaches in oral squamous cell carcinoma diagnostics using liquid biopsy. Periodontol 2000. 2024.
6. Alvarez-Errico D. Perspectives on Epigenetics and Cancer Immunotherapy: A Preface to Special Issue. Cancers (Basel). 2021;13(6).
7. Kao KC, Vilbois S, Tsai CH, Ho PC. Metabolic communication in the tumour-immune microenvironment. Nat Cell Biol. 2022;24(11):1574-1583.
8. DeNardo DG, Ruffell B. Macrophages as regulators of tumour immunity and immunotherapy. Nat Rev Immunol. 2019;19(6):369-382.
9. Zhang X, Hu Y, Cui M, et al. Cell diversity and immune infiltration in the parathyroid tumour microenvironment. Endocr Relat Cancer. 2023;30(3).
10. Gonzalez H, Hagerling C, Werb Z. Roles of the immune system in cancer: from tumor initiation to metastatic progression. Genes Dev. 2018;32(19-20):1267-1284.
11. Zhang Y, Lin L, Wu Y, Bing P, Zhou J, Yu W. Upregulation of TIMM8A is correlated with prognosis and immune regulation in BC. Front Oncol. 2022;12:922178.
12. Jiang X, Wang J, Deng X, et al. Role of the tumor microenvironment in PD-L1/PD-1-mediated tumor immune escape. Mol Cancer. 2019;18(1):10.
13. Wang D, DuBois RN. Immunosuppression associated with chronic inflammation in the tumor microenvironment. Carcinogenesis. 2015;36(10):1085-1093.
14. Cable J, Greenbaum B, Pe'er D, et al. Frontiers in cancer immunotherapy-a symposium report. Ann N Y Acad Sci. 2021;1489(1):30-47.
15. Chi H, Jiang L, Zhang S, Liu Y, Yang G, Tian G. Editorial: Targeting key cellular signaling network for cancer chemotherapy and immunotherapy. Front Immunol. 2024;15:1425261.
16. Riley RS, June CH, Langer R, Mitchell MJ. Delivery technologies for cancer immunotherapy. Nat Rev Drug Discov. 2019;18(3):175-196.
17. Matsuura V, Yoshida CA, Komori H, et al. Expression of a Constitutively Active Form of Hck in Chondrocytes Activates Wnt and Hedgehog Signaling Pathways, and Induces Chondrocyte Proliferation in Mice. Int J Mol Sci. 2020;21(8).
18. Chen CL, Chien SC, Leu TH, Harn HI, Tang MJ, Hor LI. Vibrio vulnificus MARTX cytotoxin causes inactivation of phagocytosis-related signaling molecules in macrophages. J Biomed Sci. 2017;24(1):58.
19. Khella CA, Franciosa L, Rodirguez-Rodriguez L, Rajkarnikar R, Mythreye K, Gatza ML. HCK Promotes High-Grade Serous Ovarian Cancer Tumorigenesis through CD44 and NOTCH3 Signaling. Mol Cancer Res. 2023;21(10):1037-1049.
20. Poh AR, Dwyer AR, Eissmann MF, et al. Inhibition of the SRC Kinase HCK Impairs STAT3-Dependent Gastric Tumor Growth in Mice. Cancer Immunol Res. 2020;8(4):428-435.
21. Carvalho MFL, de Almeida BO, Bueno MLP, et al. Comprehensive analysis of the HCK gene in myeloid neoplasms: Insights into biological functions, prognosis, and response to antineoplastic agents. Hematol Transfus Cell Ther. 2023.
22. Lantermans HC, Ma F, Kuil A, et al. The dual HCK/BTK inhibitor KIN-8194 impairs growth and integrin-mediated adhesion of BTKi-resistant mantle cell lymphoma. Leukemia. 2024.
23. Xia S, Li JD, Yan SB, et al. Clinicopathological value of hematopoietic cell kinase overexpression in laryngeal squamous cell carcinoma tissues. Pathol Res Pract. 2023;247:154534.
24. Bottema RW, Postma DS, Reijmerink NE, et al. Interaction of T-cell and antigen presenting cell co-stimulatory genes in childhood IgE. Eur Respir J. 2010;35(1):54-63.
25. Cha YJ, Kim EY, Choi YJ, Kim CY, Park MK, Chang YS. Accumulation of plasmacytoid dendritic cell is associated with a treatment response to DNA-damaging treatment and favorable prognosis in lung adenocarcinoma. Front Immunol. 2023;14:1154881.
26. Hotter D, Sauter D, Kirchhoff F. Emerging role of the host restriction factor tetherin in viral immune sensing. J Mol Biol. 2013;425(24):4956-4964.
27. Gao Q, Mo S, Han C, et al. Comprehensive analysis of LILR family genes expression and tumour-infiltrating immune cells in early-stage pancreatic ductal adenocarcinoma. IET Syst Biol. 2023;17(2):39-57.
28. Wu Y, Ni H, Yang D, et al. Driver and novel genes correlated with metastasis of non-small cell lung cancer: A comprehensive analysis. Pathol Res Pract. 2021;224:153551.
29. Tian S, Luo M, Liao X, et al. Integrated immunogenomic analysis of single-cell and bulk profiling reveals novel tumor antigens and subtype-specific therapeutic agents in lung adenocarcinoma. Comput Struct Biotechnol J. 2024;23:1897-1911.
30. Luo Q, Hu S, Tang Y, Yang D, Chen Q. PPT1 Promotes Growth and Inhibits Ferroptosis of Oral Squamous Cell Carcinoma Cells. Curr Cancer Drug Targets. 2024.
31. Sharma G, Ojha R, Noguera-Ortega E, et al. PPT1 inhibition enhances the antitumor activity of anti-PD-1 antibody in melanoma. JCI Insight. 2022;7(20).
32. Weng J, Liu S, Zhou Q, et al. Intratumoral PPT1-positive macrophages determine immunosuppressive contexture and immunotherapy response in hepatocellular carcinoma. J Immunother Cancer. 2023;11(6).
33. Harding JJ, Awada A, Roth G, et al. First-In-Human Effects of PPT1 Inhibition Using the Oral Treatment with GNS561/Ezurpimtrostat in Patients with Primary and Secondary Liver Cancers. Liver Cancer. 2022;11(3):268-277.
34. Jia Y, Zhang Y, Zhu H. Clindamycin Derivatives: Unveiling New Prospects as Potential Antitumor Agents. Pharmaceuticals (Basel). 2024;17(3).
35. Fabian KP, Malamas AS, Padget MR, et al. Therapy of Established Tumors with Rationally Designed Multiple Agents Targeting Diverse Immune-Tumor Interactions: Engage, Expand, Enable. Cancer Immunol Res. 2021;9(2):239-252.

**Supplementary Figures**


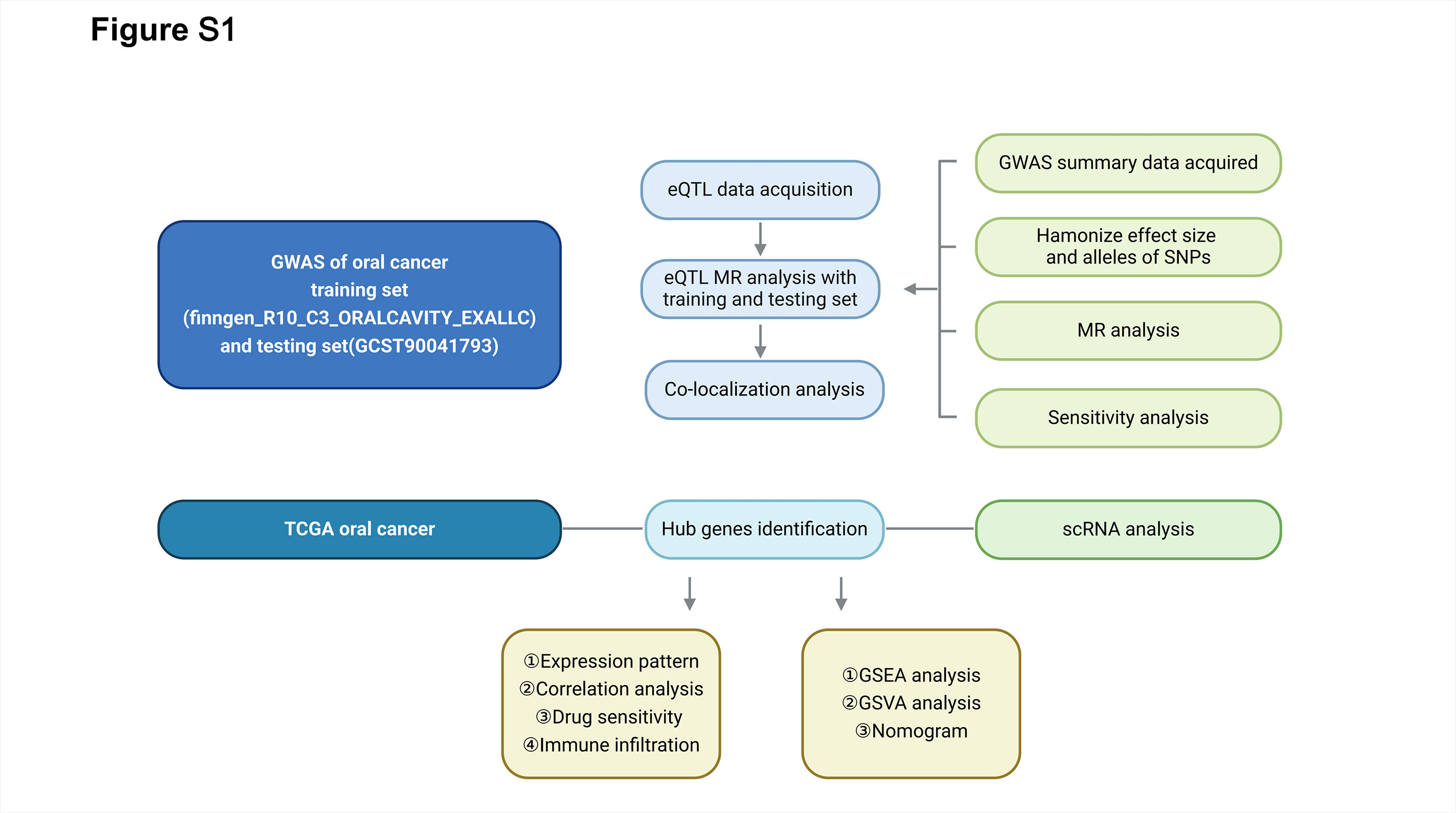


**Figure S1.** Illustration of the study design schematic.


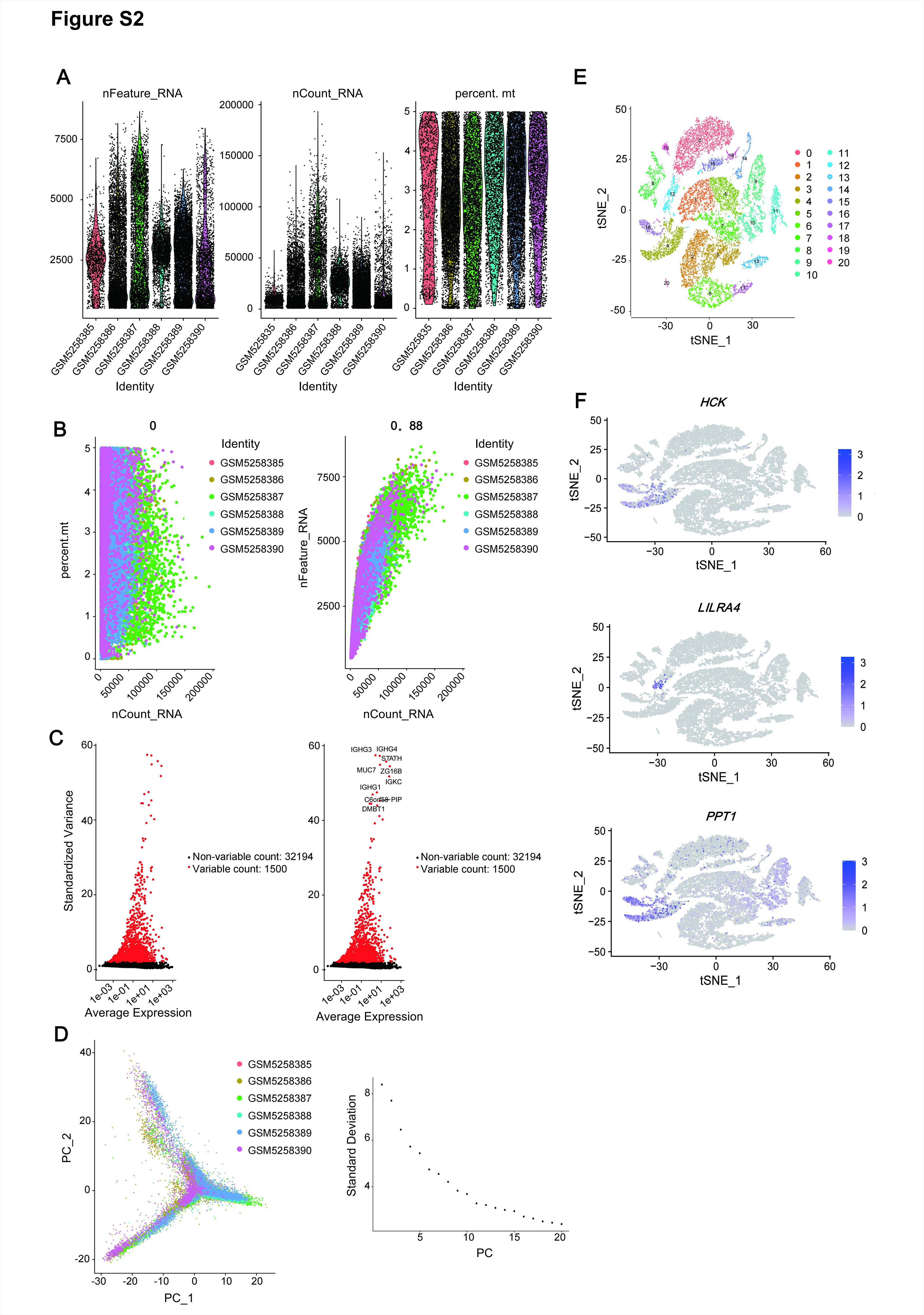


**Figure S2.** Single-cell pre-processing and the expression profile. (A) A single-cell quality assessment procedure, including cell count, gene count, as well as sequencing depth in each sample. (B) The graphs respectively showed the correlation between sequencing depth and mitochondrial content or gene count, with both being positively correlated. (C) Identification and visualization of genes with significant differences between cells, highlighting characteristic variance. (D) PCA and PC distribution, with dots representing cells and colors denoted samples. Variance ranking plot. (E) We employed the t-SNE algorithm to categorize the cells into 21 clusters in accordance with significant components derived from PCA. (F) Expression of the critical genes in single cells.


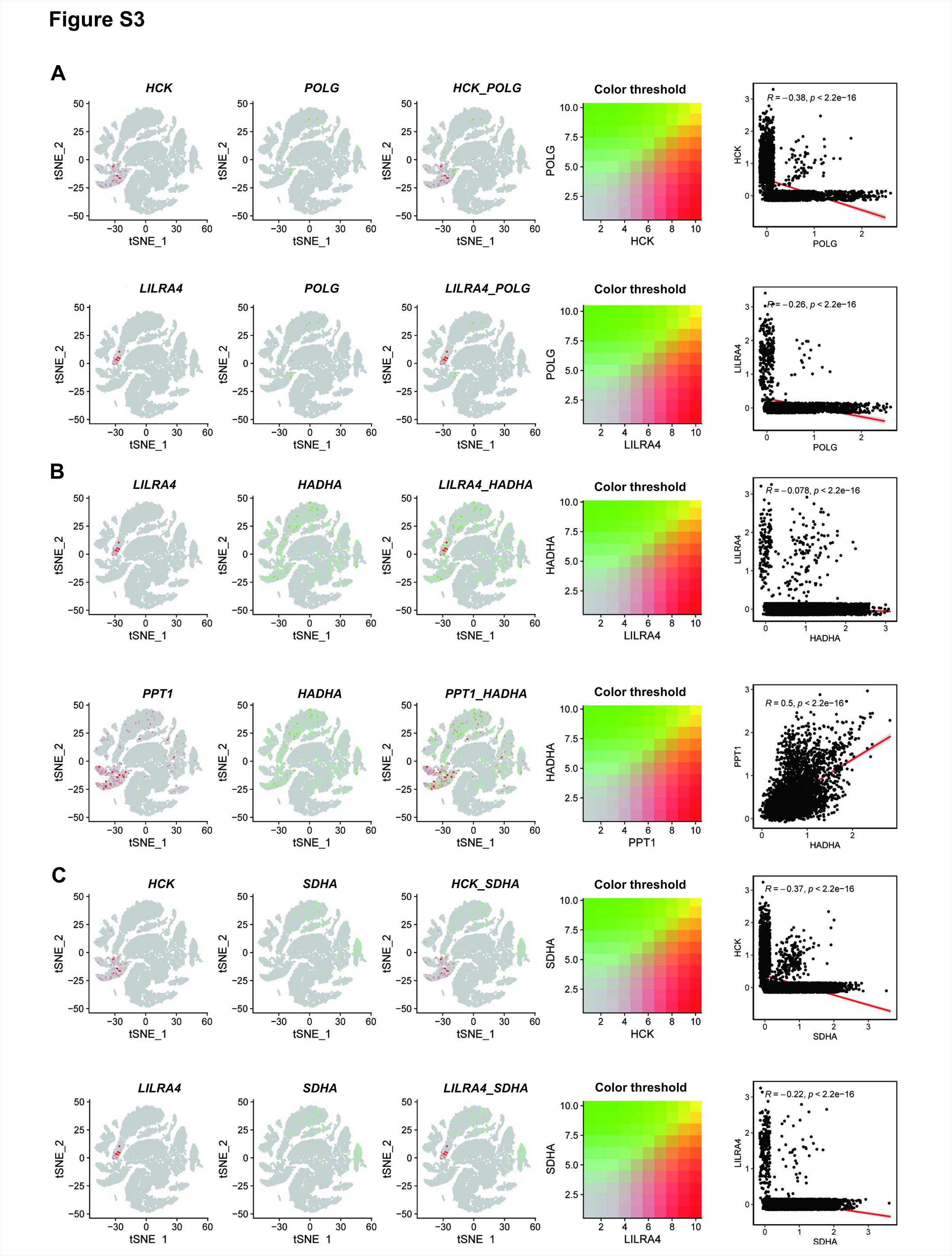


**Figure S3**. Gene co-expression of mitochondria-related genes with key genes in single-cell data, and the co-expressed gene correlations. (A) Mitochondria-related gene *HADHA*. (B) Mitochondria-related gene *POLG*. (C) Mitochondria-related genes *SDHA*.


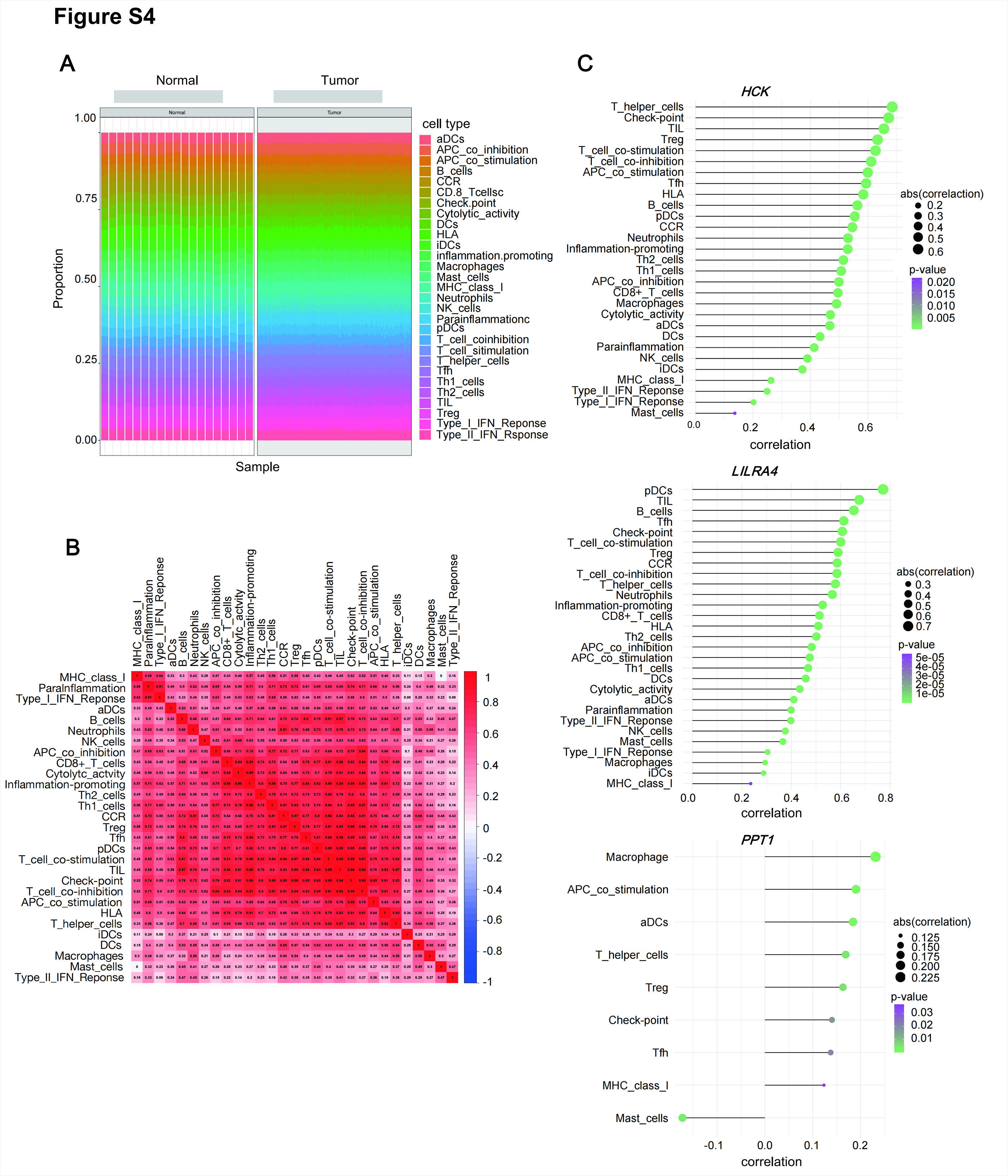


**Figure S4.** Immune infiltration analysis. (A) Relative proportions of 29 immune cell subsets. (B) The graph of Pearson correlation among the 29 immune cell types, where negative correlations were shown in blue and positive correlations in red. (C) Comparative analysis of immune cell content in normal samples versus tumor samples.


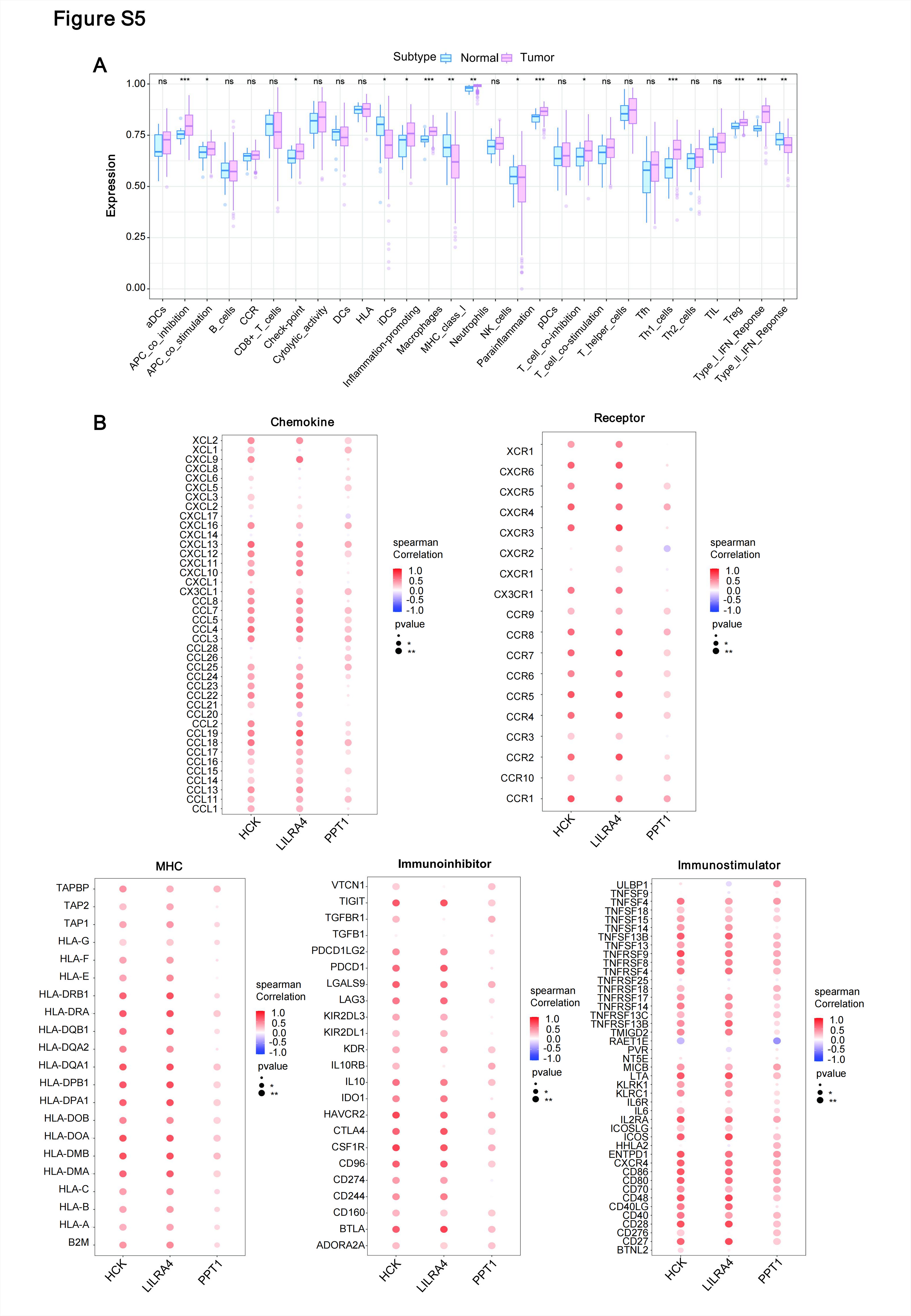


**Figure S5.** Interactions of Immune-related genes and cells (A) Association of the key genes with immunity and immune cells. (B) Interactions between critical genes and immune-related factors.

**
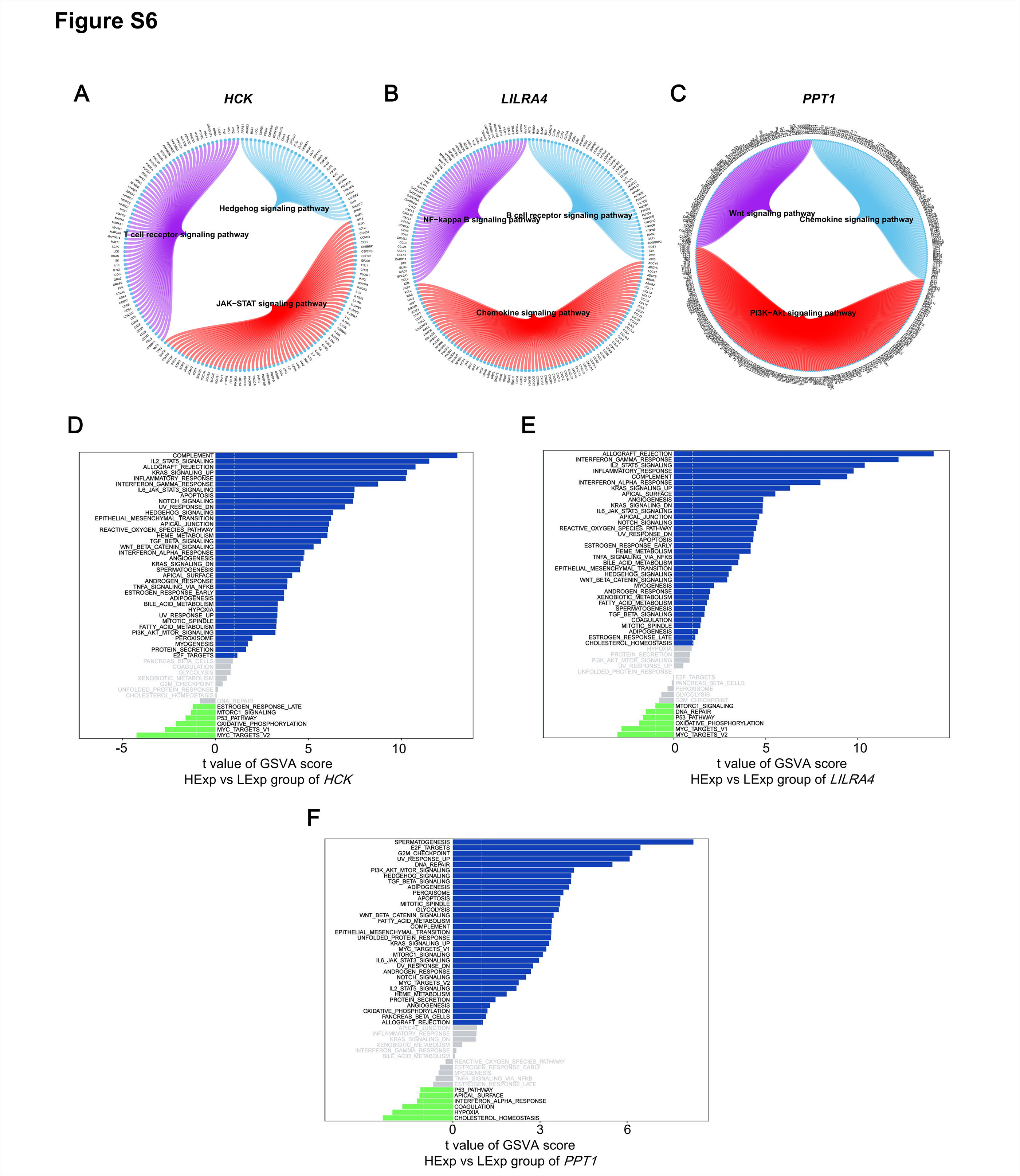
**

**Figure S6.** GSEA and GSVA analyses. (A-C) The main KEGG signaling pathways of the critical genes involved. (D-F) Results of the GSVA analysis. Blue indicated pathways linked to high gene expression, green indicated pathways linked to low gene expression, with the hallmark gene set as the reference.
